# Evidence and Mechanisms of Fat Depletion in Cancer

**DOI:** 10.3390/nu6115280

**Published:** 2014-11-19

**Authors:** Maryam Ebadi, Vera C. Mazurak

**Affiliations:** Division of Human Nutrition, Department of Agricultural, Food and Nutritional Science, University of Alberta, 4-002 Li Ka Shing Centre for Health Research Innovation, Edmonton, AB T6G 2E1, Canada; E-Mail: ebadi@ualberta.ca

**Keywords:** adipose tissue, cancer, computed tomography, fat mobilization

## Abstract

The majority of cancer patients experience wasting characterized by muscle loss with or without fat loss. In human and animal models of cancer, body composition assessment and morphological analysis reveals adipose atrophy and presence of smaller adipocytes. Fat loss is associated with reduced quality of life in cancer patients and shorter survival independent of body mass index. Fat loss occurs in both visceral and subcutaneous depots; however, the pattern of loss has been incompletely characterized. Increased lipolysis and fat oxidation, decreased lipogenesis, impaired lipid depositionand adipogenesis, as well as browning of white adipose tissue may underlie adipose atrophy in cancer. Inflammatory cytokines such as interleukin-6 (IL-6), tumor necrosis factor alpha (TNF-α), and interleukin-1 beta (IL-1β) produced by the tumor or adipose tissue may also contribute to adipose depletion. Identifying the mechanisms and time course of fat mass changes in cancer may help identify individuals at risk of adipose depletion and define interventions to circumvent wasting. This review outlines current knowledge of fat mass in cancer and illustrates the need for further studies to assess alterations in visceral and subcutaneous adipose depots and possible mechanisms for loss of fat during cancer progression.

## 1. Introduction

Adipose tissue (AT) is an active secretory organ that regulates energy balance, homeostasis, appetite, inflammation, insulin sensitivity, angiogenesis, and fat metabolism [1]. Adipose tissue metabolism and whole body fat mass are regulated through two major pathways: lipolysis (fat breakdown) and lipogenesis (fat synthesis) [2]. Adipose tissue dysfunction, fat mass changes and concurrent alterations in the production of adipokines, inflammatory cytokines, and lipid metabolites are common in metabolic disorders, such as insulin resistance, type 2 diabetes, cardiovascular disease, and obesity-related cancers such as colorectal and breast cancer [3,4].

The link between obesity and increased cancer incidence is well established [5,6], but the relationship between fat mass and cancer progression is much less clear. Studies indicate that the majority of cancer patients experience some degree of cancer-related wasting of both muscle and/or fat during the disease trajectory [7]. However, little is known about the importance of fat loss in cancer because the majority of studies of cancer-associated wasting typically focus on muscle. Potential links between fat loss and poor outcomes have been identified that indicate fat loss to be a poor prognostic factor in advanced cancer regardless of a patient’ body weight [8,9]. This article reviews current knowledge of adipose tissue depletion in cancer, focusing on both assessment of fat tissue and morphological determination of adipose tissue in cancer populations. Possible mechanisms of fat loss are also discussed. Biological alterations in adipose tissue metabolism precede the physical manifestation of adipose tissue loss. Thus, understanding mechanisms and potential markers of fat loss in cancer are important for early detection which facilitates prevention of further loss to preserve fat and improve survival in cancer patients.

## 2. Adipose Atrophy in Cancer

Fat loss has been reported to be associated with shorter survival time [8,9]. Analysis of adipose tissue morphology and body composition has revealed body fat depletion in human and animal models of cancer cachexia. In the majority of human studies discussed in this review, cachexia is defined as ≥5% weight loss (WL) over 3 months or ≥10% within the previous 6 months. Weight loss does not necessarily reflect the severity of cachexia and fat loss but is the first outcome measurement typically used in studies of cancer. Validated data for classification of cachexia based on recent consensus are emerging [10].

The murine adenocarcinoma (MAC16) causes diminished adipocyte size with increased mitochondrial density, and elevated adipose tissue fibrosis in cachectic mice, compared to pair-fed and control animals [11]. The Walker 256 carcinoma, a well-established cancer cachexia model, affects adipose tissue in a time and depot-dependent manner [12,13,14]. Seven days after Walker 256 tumor injection, no significant changes were observed in adipocyte size. However, after 14 days, adipocyte size of retroperitoneal, and epididymal adipose tissue was decreased. On the other hand, mesenteric adipose tissue was not lost and size of mesenteric adipocytes increased after 14 days [13,14]. In support of these experimental studies, reduction in fat cell volume has been reported in weight-losing gastrointestinal (GI) cancer patients [15,16,17]. Cachectic patients exhibited smaller adipocytes compared to weight-stable controls [15,16,17,18] and non-cancer patients [16] but total body fat cell number was not altered [16,17]. Collectively, these studies suggest altered adipocyte size and reduced lipid storage capacity in the presence of a tumor.

## 3. Assessment of Fat Tissue over the Cancer Trajectory

The body mass index (BMI) has been used frequently as a clinically accessible measure of human body composition. However, the BMI does not differentiate between fat and fat-free mass or fat depots. Accumulation or loss of specific fat tissues are differentially associated with health outcomes. For example, there is a relationship between visceral adipose tissue (VAT) accumulation and insulin resistance [19]. Insulin resistant adipocytes that reside in VAT are more sensitive to catecholamine-induced lipolysis than subcutaneous adipose tissue (SAT) [1,20]. Lipolysis of fat from VAT enables direct delivery of free fatty acids to liver, which may lead to elevated hepatic triglyceride (TG) production, increased very low density lipoprotein secretion, and higher plasma TGs which exacerbates an already dysregulated metabolic state [20]. Therefore, an understanding of the intensity of loss and the type of fat being lost (VAT *vs.* SAT) is required. Potential differences in fat loss between depots has not been consistently demonstrated, partly due to the use of analytical techniques with limited applicability, as well as the variability among studies with regards to tumor type, stage and the time-point in the cancer trajectory that patients are studied. Discrepant methods of assessing fat and reporting values as cross sectional area (cm^2^) or volume (cm^3^), total fat mass (kg or %), change in area or the rate of changes also limit the ability to interpret and compare studies.

Body composition is assessed in cancer patients using a variety of methods including bioelectrical impedance analysis (BIA), dual-energy X-ray absorptiometry (DEXA), magnetic resonance imaging (MRI) and computed tomography (CT) scan analysis [21]. Body composition analysis using BIA has demonstrated lower body fat (% or kg) in cachectic patients compared to weight-stable cancer controls [15,16,17,22], healthy controls [23], or non-malignant controls [16,22]. When DEXA was applied to malnourished palliative cancer patients, no differences were observed in absolute fat mass (kg) during follow-up (4–62 months) [9]. However, the relative change (percentage of change from initial values) revealed a loss of fat concurrent with a marginal increase in lean mass during cancer progression [9]. As DEXA quantifies regional lean body mass, this study raised the possibility that patients may not have been gaining skeletal muscle per se but rather lean mass in internal organs such as the liver and spleen which has been reported as patients approached death in a subsequent study [24].

In an oncologic population, CT images are a routine part of treatment and are available from patient records as a chart review. CT image analysis has emerged as the gold standard for body composition assessment in cancer patients due to its ability to discriminate and quantify muscle, adipose tissue and organs. Shen *et al.* established that single slice tissue areas can be used to estimate whole body muscle and adipose tissue volumes [25]. Cachectic cancer patients exhibit lower adipose tissue mass compared to weight stable and/or controls [15,16,17,26]. Volumes of total adipose tissue, VAT and SAT were calculated in newly diagnosed GI cancer patients receiving no anticancer treatment [26]. Cachectic groups were separated into two groups, those with and without gastrointestinal obstruction that interfered with their food intake. Cachectic groups were compared to weight-stable cancer patients. Deterioration in nutritional status was confirmed by a higher Patient-Generated Subjective Global Assessment (PG-SGA) score in the cachectic patients with GI obstruction. Both BIA and CT analysis indicated that total fat mass (kg), and visceral and abdominal subcutaneous volumes were lower in cachectic patients compared to the weight-stable group. The cachectic patients with GI obstruction lost approximately two times more weight but VAT volume was greater compared to cachectic group without GI obstruction [26]. This study applied CT scans taken at one point in time; therefore, intensity of loss over time can not be determined. A lower amount of VAT, not the loss per se, was observed in cachectic group who did not have altered food intake.

Approaching death, the intensity of tissue loss increases and patients experience the greatest and most accelerated rate of loss [8,9,24,27]. Analysis of sequential CT images in 34 advanced colorectal cancer patients revealed that the greatest changes in body composition occur starting at 4.2 months from death [24]. One month from death, liver and spleen mass increase, whereas skeletal muscle and fat mass decrease [24]. A study by Murphy *et al.* quantified fat mass in 108 colorectal and lung cancer patients with at least two abdominal CT images in the last 500 days of life. Beginning seven months prior to death, both VAT and SAT mass decreased in cancer patients, reaching intensities of 10 kg of fat loss/100 days [8]. A recent study in pancreatic cancer patients suggested that the rate of visceral adipose tissue loss, rather than the absolute amount, may be an important indicator of survival [28]. Patients with at least two abdominal CT scans between diagnosis and death, receiving surgery (62%) or chemotherapy (88%) during cancer progression, were selected for this study. The rate of change (% change/100 days) for SAT was similar to VAT but a change in VAT was significantly correlated with survival in cancer patients. The presence of co-morbidities such as diabetes and anemia may have accelerated loss of VAT [28]. In another study, cachectic gastrointestinal cancer patients had significantly lower VAT and SAT three months prior to death compared to the benign controls. However, there was a tendency for cachectic patients to have smaller visceral and subcutaneous area compared to cancer patients without cachexia [27]. At present, no other studies exist regarding the pattern of fat loss in cancer and further studies are needed to establish the timeline and pattern of fat mass alterations in different adipose tissue depots during cancer progression. Further, the majority of studies assessing fat mass focus on gastrointestinal cancer patients so there is a gap in knowledge related to other malignant tumors.

**Figure 1:**
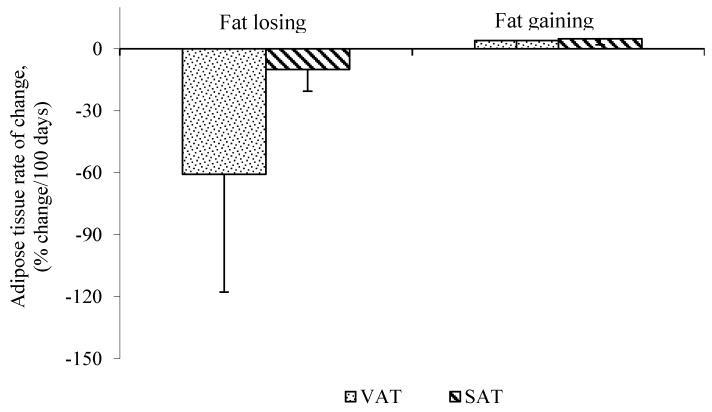
Mean rate of visceral adipose tissue (VAT) and subcutaneous adipose tissue (SAT) change in fat losing cancer patients assessed by consecutive computed tomography (CT) scans. Data are represented as Mean ± SD, *n* = 5 (Fat Losing) and *n* = 2 (Fat Stable), *p* < 0.05 VAT, visceral adipose tissue; SAT, subcutaneous adipose tissue.

While the majority of human studies focus on cachectic *vs.* non-cachectic patients, less is known about the effect of cancer treatments, which may also induce alterations in fat mass. For example, cancer surgery contributes to weight loss. Six months after surgery, weight is reduced from the baseline due to the catabolic response to the operation [29,30,31] and stabilizes after 12 months [31]. Adams reported that weight loss occurs rapidly in the three months following surgery [29]. Body composition assessment before, and 6 and 12 months after gastrectomy, measured from total body potassium and water, indicated 40% of fat mass was lost during the six months after surgery [30]. In a study by our group, two to six months after surgery, patients with colorectal liver metastasis were losing VAT at a greater rate than SAT, measured using consecutive CT scans (Figure 1). This supports the work of others in pancreatic cancer patients during early stages of disease progression. Intra-abdominal and subcutaneous adipose tissue mass were assessed before and after surgery using CT scans. Fat loss from intra-abdominal depot was greater than abdominal subcutaneous following surgery [32]. Therefore, surgical procedures may contribute to weight and fat loss due to the catabolic and inflammatory response to the surgery.

Fat loss or gain after chemotherapy will depend on the tumor type, drug type, dose and overall response to chemotherapy. Following at least one cycle of chemotherapy treatment (cisplatin, 5-fluorouracil and/or epirubicin), patients with locally advanced oesophagogastric cancers lost an average of 1.3 ± 3.2 kg (6%) fat mass [33]. In advanced pancreatic cancer patients, a multivariate survival analysis revealed that VAT loss (determined from CT pre and post chemoradiation) but not muscle loss was significantly related to shorter survival [34].

Three months after chemotherapy initiation, testicular cancer patients who received 3 or 4 cycles of cisplatin-based chemotherapy had significantly higher VAT volume without changes in SAT [35]. However, nine months later, both VAT and SAT increased significantly, suggesting a capacity to rebuild lost adipose tissue [35]. A recent study using CT imaging to understand the loss and gain of muscle and adipose tissue during the year preceding death revealed that anabolic potential does exist, as some patients gained muscle and adipose tissue, but were only capable of doing this >3 months prior to death [36]. These results will initiate further research aiming to define the appropriate time to initiate nutritional intervention to preserve both muscle and fat tissue.

Fat loss may precede the loss of lean tissues (Table 1). The only patient group in which this question has been addressed is patients with newly diagnosed GI cancers. However, in all studies that have addressed this question to date, changes in adipose tissue were observed in absence of changes in lean tissues. The majority of these studies use BIA and DEXA for body composition assessments which are limited in ability to provide a direct estimate of muscle mass; further studies are needed to confirm that these findings are attributable to muscle loss or other lean body mass loss. Only one study used CT scans to assess body composition in GI cancer patients and that study showed no difference in abdominal muscle volume between cachectic and weight-stable cancer patients. However, that study assessed CT images at only one time point [26]. Adipose depletion may occur more rapidly than muscle during disease progress. Advanced pancreatic cancer patients lost both VAT and SAT over time, and the rate of change (%change/100 days) in total adipose tissue (−40.4 ± 25.4%/100 days) was much greater than muscle tissue (−3.1 ± 12.0%/100 days). No significant differences in adipose tissue mass were observed between patients who were or were not receiving chemotherapy [37]. These observations are supported by an experimental study in which lung carcinoma or melanoma cells were injected subcutaneously to induce cachexia in mice. Fat loss occurred prior to muscle loss, at early stages of tumor growth, at an intensity that was greater than muscle loss [38]. White adipose tissue browning, which contributes to fat loss in cancer, occurred before skeletal muscle wasting in mouse models of cancer cachexia [39].

**Table 1:** Articles reporting fat and lean tissue loss in newly diagnosed cancer patients.

Authors	Subjects ^1^	Cancer Type	Body Composition Assessment	General Comments
Fouladiun *et al.*, [9]	Malnourished patients (*n* = 132; 66 ± 3 years) advanced cancer with malnutrition (T4N1M1)	GI (*n* = 123) Breast (*n* = 1) Melanomas (*n* = 2) Other (*n* = 6), followed for 6–42 months	DEXA	Whole body fat loss was related to shorter survival Body fat loss more intense and pronounced compared to lean tissue
Agusstson *et al.*, [15]	Weight stable cancer patients (*n* = 11), Weight-losing cachectic cancer patients with (*n* = 8) and without (*n* = 7) malnutrition	GI cancer with no treatment before surgery	BIA	No differences in lean body mass between groups Increased lipolysis in cancer cachectic patients
Dahlman *et al.*, [17]	Cachectic patients (*n* = 13) Weight-stable cancer (*n* = 14)	GI cancer with no treatment before surgery	BIA	Decreased body fat mass but similar lean body mass between cachectic and control patients
Ryden *et al.*, [16]	Cachectic patients (*n* = 13) Weight stable cancer patients (*n* = 10), Without cancer (*n* = 5)	GI cancer with no treatment before surgery	BIA	No difference in lean body mass between groups Elevated lipolysis with no changes in lipogenesis No local inflammation
Agustsson *et al.*, [26]	Cancer cachectic without (*n* = 13) and with gastrointestinal obstruction (*n* = 10), Weight losing-cancer (*n* = 17)	GI cancer with no treatment before surgery	BIA, CT	No changes were observed in lean mass Visceral fat volume was lower in cachectic group compared to weight stable

^1^ No patients received chemotherapy or radiotherapy.

## 4. Mechanisms for Adipose Depletion in Cancer

**Figure 2:**
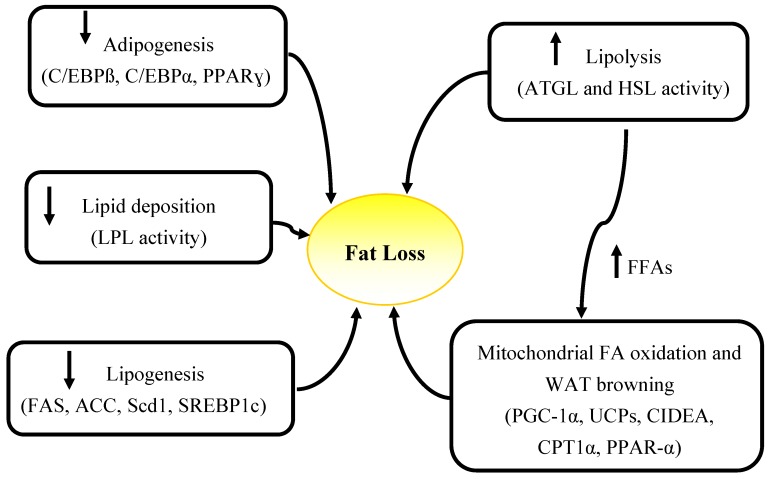
Summary of mechanisms and specific genes involved in adipose atrophy in cancer. WAT, white adipose tissue; FFAs, free fatty acids; ATGL, adipose triglyceride lipase, HSL, hormone sensitive lipase; PGC-1α, peroxisome proliferator-activated receptor-gamma coactivator 1 alpha; UCPs, un-coupling proteins; CIDEA, Cell death-inducing DFFA (DNA fragmentation factor-alpha)-like effector A; CPT1α, carnitine palmitoyltransferase 1 alpha; PPAR-γ, Peroxisome proliferator-activatedreceptor gamma; C/EBPα, CCAAT-enhancer-binding proteinα; LPL, lipoprotein lipase; FAS, fatty acid synthase; ACC, Acetyl-CoA carboxylase; Scd1, Stearoyl-CoA desaturase; SREBP1c, sterol regulatory element binding protein-1c.

Elevated energy expenditure, decreased food intake and alterations in circulating levels of hormones including insulin, leptin, catecholamines, as well as elevated catabolism due to the tumor presence (high energy demands of tumor, inflammatory mediators produced by tumor) and tumor-host interactions are factors contributing to wasting in cancer [40]. These factors can cause abnormalities in lipid metabolism which may also lead to fat loss. Increased lipolytic activity, evidenced by elevated fasting plasma glycerol and free fatty acids is a driver of fat loss in advanced cancer patients [15,23,41] but the underlying causes of elevated lipolysis are not known. Other mechanisms including decreased lipogenesis [42,43], impairment in adipogenesis [11,14], elevated fat oxidation [17,23,44], and decreased lipid deposition [45,46,47,48,49] have also been attributed to fat loss in cancer (Figure 2).

Human and experimental models have been used to study the mechanisms of fat loss in cancer. Animal models are necessary to elevate our understanding of cancer associated weight-loss. However, each model may represent only some aspects of human cancer cachexia and choice of animal model is based on research objectives. For example, the MAC16 adenocarcinoma induces cachexia in the absence of anorexia and is suitable to study wasting related to the tumor produced factors rather than food intake. Yoshida ascites hepatoma AH130 (YAH-130), on the other hand, induces cachexia and anorexia accompanied by inflammation [50]. Therefore, the result of studies investigating the mechanisms underlying fat loss in cancer should be interpreted with caution as each specific tumor type, in various stages of growth, can affect various adipose tissue depots in a different manner.

### 4.1. Decreased Food Intake and Hypermetabolism

Anorexia alone does not explain reduced body weight or/and fat mass in cancer patients and cachexia-associated wasting can not be completely reversed by elevated nutritional intake [10]. Compared to pair-fed controls, the MAC-16 tumor leads to greater fat loss in mice indicating that tumors with high energy demands, rather than calorie restriction, may be responsible for adipose depletion [11]. Likewise, human studies have shown that in the absence of changes in food intake, hyper metabolism, characterized by elevated resting energy expenditure (REE), may be a contributing factor to the weight loss in cancer. Weight-losing and weight-stable cancer patients with various solid tumors had similar dietary intakes but weight losing patients had a higher REE determined by indirect calorimetry [51]. REE was also higher in weight-stable cancer patients compared to non-cancer controls which indicate that the tumor contributes to an elevated REE [52]. Johnson *et al.* reported no difference in measured REE between weight-losing and stable cancer patients but rather attributed higher REE in weight-losing cancer patients to elevated C-reactive protein (CRP) [53]. Although REE can contribute to weight loss in cancer, factors like tumor type, stage and duration of the disease also affect the REE in cancer [52,54].

In palliative cancer patients, during 4–62 months follow-up, body weight and fat mass (% change from baseline) decreased in the absence of changes in REE. Despite providing nutritional support to patients who had baseline calorie intake less than 90% of their energy requirement, body weight and fat mass did not increase [9]. Therefore, factors other than nutrient intake and hypermetabolism may contribute to fat loss in cancer.

### 4.2. Lipolysis and Elevated Fat Oxidation

It is well accepted that elevated lipolysis is the main cause of fat loss in cancer [15,16,17,22,23,55,56], however the specific mechanisms contributing to lipolysis have not been clearly defined. Hormone sensitive lipase (HSL) and adipose triglyceride lipase (ATGL) are major enzymes that contribute to TG breakdown in adipose tissue. Adipose triglyceride lipase catalyzes the first step in TG hydrolysis. During adipose tissue lipolysis, free fatty acids (FFA) and glycerol molecules are produced by the hydrolysis of triglyceride. HSL activity is regulated by hormones, *i.e.*, catecholamines, insulin and glucagon, through a cAMP-mediated process [57,58]. Catecholamines stimulate lipolysis, whereas insulin has anti-lipolytic functions [59]. Binding of hormones to G-protein-coupled receptors results in up-regulation of adenylate cyclase which leads to an increase in intracellular cyclic adenosine monophosphate (cAMP) concentrations. cAMP stimulates a protein kinase which in turn phosphorylates and activates HSL [57,58]. Phosphorylated HSL translocates from the cytosol to the surface of lipid droplets to induce lipolysis.

Elevated expression of HSL mRNA [15,22,60] and protein [15,22] has been reported in cancer cachectic patients compared to weight-stable cancer. Higher mRNA expression of HSL in SAT was associated with higher serum FFAs in cancer patients, however, no significant differences were observed in mRNA expression of lipoprotein lipase (LPL), fatty acid synthase (FAS), insulin and tumor necrosis factor alpha (TNF-α) in adipose tissue of cancer patents compared to controls [60]. These results are supported by a study that reported HSL mRNA and protein over-expression, as well as increased hormone-stimulated lipolysis in cachectic cancer patients compared to malnourished weight-losing and weight stable cancer patients, explained the elevated adipose atrophy in cancer cachexia [15]. The ratio of plasma glycerol/body fat (index of *in vivo* lipolysis) was two times higher in cachectic patients. *Ex vivo* culture of adipocytes from the same patients revealed no difference in basal lipolysis (glycerol release to the media) between groups. However, incubation of adipocytes with catecholamines and natriuretic peptides elevated glycerol release to the media in the cachectic group suggesting that the adipocytes were more sensitive to the same amount of stimuli, and therefore more catabolic. There was no significant difference in plasma levels of catecholamines and natriuretic peptides between groups [15]. An explanation of the lack of difference in plasma hormone levels could be that lipolytic effects of hormones are elevated at the receptor level, evidenced by elevated β1-adrenoceptor (ADRB1) expression on adipocyte membranes in cachectic GI cancer patients [22]. Consequently, higher HSL expression and activity, which positively correlated with ADRB1 expression, were associated with higher plasma glycerol/fat mass and FFA/fat mass [22]. Therefore, lipolysis can be elevated in cancer cachectic patients due to increased expression of receptors on adipocytes membrane and their response to lipolytic effects of hormones, rather than elevated levels of mediators.

In study by Agustsson *et al.* [15], elevated HSL mRNA and protein expression contributed to the increased lipolysis. No significant difference in mRNA expression of ATGL was observed between cachectic cancer patients and controls; however, protein expression was not measured in this study [15]. Das and Hoefler [61] reported that ATGL mRNA expression may not translate to enzyme activity as its function is regulated via post-translational modifications. In another study, Das *et al.* reported higher ATGL and HSL activity in VAT of cachectic patients compared to non-cancer and cancer patients without cachexia, which has been previously reported [38]. Animal studies suggest that ATGL plays a more important role in adipose tissue lipolysis than HSL [38,62]. In mice bearing cachexia-inducing lung carcinoma or melanoma cells, lower body weight, decreased fat and muscle mass and elevated lipolysis were observed in tumor group compared to the controls. In HSL deficient mice, the tumor could reduce body weight and fat mass due to elevated ATGL activity. However, in ATGL deficient mice, the tumor did not induce elevated lipolysis and there was no significant difference in weight and fat mass between control and tumor group. Fat preservation in ATGL deficient mice, prevented muscle loss in tumor bearing animals [38]. Consistent with these findings, a recent study in mice bearing cachectic Colon-26 carcinoma revealed lower fat mass and increased lipolysis in cachectic mice compared to control mice. Increased lipolysis was induced by ATGL rather than the PKA/HSL pathway during late stages of cancer cachexia. ATGL protein levels increased in cachectic mice, however, no changes were observed in ATGL mRNA expression [62]. Therefore, not only mRNA expression but also protein expression and/or activity of these enzymes need to be determined in adipose tissue to investigate mechanisms that underlie elevated lipolysis.

The majority of studies indicated elevated lipolysis as a reason for fat loss in cancer; and consequently, increasing fatty acid oxidation could be a tentative approach to utilize surplus fatty acids (FAs). By increasing fatty acid oxidation within adipose tissue, liberated FAs are oxidized and can not be re-esterified into TG. Zuijdgeest-van Leeuwen *et al.* [23] reported higher lipolysis and reduced food intake in weight-losing cancer patients compared to healthy weight-stable adults. Whole body lipolysis and fatty acid oxidation were higher in cancer patients compared to healthy subjects, even after adjusting for food intake. However, this heterogeneous population of cancer patients had varying degrees of weight loss (5.3%–25%/6 months) and were at different stages in the disease trajectory (1–180 months since diagnosis) [23]. Up-regulation of genes involved in mitochondrial fat oxidation such as peroxisome proliferator-activated receptor-gamma coactivator 1 alpha (PGC-1α), and uncoupling protein 2 (UCP-2) have been demonstrated in animal models of cancer cachexia [11]. Enhanced fat oxidation reflected by a decreased respiratory quotient (RQ) [17,44], higher expression of genes related to energy and FA metabolism pathways such as Krebs TCA cycle, oxidative phosphorylation, and FA degradation have been reported in patients with cachexia [17]. No differences were observed in expression of genes involved in fatty acid oxidation including PPARα, PGC-1α, carnitine palmitoyltransferase 1 alpha (CPT1α) in mice bearing Colon-26 carcinoma compared to controls. However, mRNA expression of peroxisomal bifunctional enzyme (Pbe), specific for peroxisomal fatty acid oxidation, was higher in cachectic mice [62]. Another study found that mRNA levels of Cell death-inducing DFFA (DNA fragmentation factor-alpha)-like effector A (Cidea), which mediates oxidation of excess FAs rather than glucose, is increased in SAT of cachectic patients. In addition, cachectic patients had lower plasma TGs, increased FAs and glycerol, and lower RQ indicating elevated FA oxidation [44]. In pancreatic cancer patients, Cidea expression was higher in intra-abdominal adipose tissue compared to subcutaneous in early stages of tumor progression. CT image analysis of these same patients revealed that patients were losing intra-abdominal fat more than subcutaneous fat [32].

Excess fatty acids from enhanced lipolysis are oxidized by mitochondria to produce energy. However, the appearance of brown adipocytes within the white adipose tissue can dissipate energy of substrate oxidation as heat through uncoupling fatty acid oxidation from ATP production by uncoupling protein-1 (UCP1) [63]. Recently, studies found that white adipose tissue browning can contribute to adipose atrophy in cancer by enhancing white fat thermogenesis [39,64]. Small adipocytes with large nuclei were observed during early stages of cachexia in SAT of mouse models of lung and pancreatic cancer. Multi-locular cells interspersed in the white adipose tissue, resembling brown adipocytes, positively stained for UCP-1 [39]. White adipose tissue browning was associated with increased expression of brown fat markers including UCP-1, PGC-1α, PPARγ and Cidea in cachectic mice compared to the controls [39,64]. β-adrenergic signaling, inflammatory cytokines like interleukin-6 (IL-6) [39] and tumor-derived parathyroid hormone-related protein (PTHrP) [64] can mediate white adipose browning by inducing expression of thermogenic genes. Blocking of these mediators might help to prevent adipose atrophy in cancer cachexia [39,64]. Collectively, lipolysis, increase fatty acid oxidation and elevated white adipose tissue thermogenesis play an important role in AT depletion in cancer.

### 4.3. Lipogenesis and Lipid Deposition

Despite the importance of lipolysis in fat loss in cancer, fat depletion can also occur when lipogenesis is limited in white adipose tissue. In murine cachectic models (Yoshida AH-130 ascites hepatoma), decreased AT lipogenesis was accompanied by an increase in liver lipogenesis and hypertriglyceridemia [42]. Decreased lipogenesis was accompanied by lower activities of FAS, citrate cleavage enzyme, and malic enzyme in rats bearing a mammary adenocarcinoma during late phases of tumor progression [43]. Deterioration in lipid synthesis capacity of epidydimal adipose tissue was observed in MAC16 bearing rats, evidenced by decreased mRNA levels of important lipogenic enzymes such as acetyl-CoA carboxylase, FAS, stearoyl-CoA desaturase-1 and glycerol-3-phosphate acyltransferase [11].

Increased lipolysis and decreased lipogenesis has been reported in male Japanese white rabbits bearing the VX2 tumor cells compared to food-restricted animals. Body weight reduction and fat loss occurred before any decrease in food intake [65]. Adipocyte apoptosis (20–30 days after tumor implantation) was also observed in tumor groups, however no changes in total body fat cell numbers has been reported in previous human studies [15,16,17]. Discrepancies may be caused by the fact that patients in previous studies were at early stages of disease and also the fat cell numbers were extrapolated based on the total body fat and mean fat cell volume. In contrast to those human studies, animals were followed during cancer progression and also biological differences and limitations of extrapolating results between different species may contribute to the discrepancies.

LPL mediate FAs uptake in adipose tissue by hydrolysis of very-low-density lipoproteins and chylomicrons. Numerous animal studies suggest reduced LPL activity in cancer [42,43,45,46]. Reduction in AT LPL activity in tumor bearing mice to the levels of starved animals was associated with impaired lipid deposition, fat loss, reduced breakdown of plasma lipoproteins and increased circulating lipid concentrations [47]. Decreased adipose tissue LPL activity was associated with hypertriglyceridemia during early stages of tumor growth in Lewis rats bearing a mammary adenocarcinoma [43]. Decreased fat content and LPL activity in WAT was accompanied by increasing circulating triglycerides, and body weight loss induced by the Yoshida AH-130 ascites hepatoma in rats [42,45,46]. In mice bearing MAC16, plasma TGs decreased during cancer progression, regardless of the amount of weight loss. At early stages, plasma FFA decreased and LPL activity increased; however, at advanced stages of tumor, LPL activity decreased [49].

While the majority of studies have utilized animal models to investigate lipogenesis and LPL activity during cancer progression, the human studies have reported decreased mRNA expression and activity of LPL and FAS in VAT in proximity to a tumor compared to distal adipose tissue in colorectal cancer patients [48]. Decreased FAS activity in adipose tissue and elevated activity in tumor cells might be important for tumor cell growth [48]. No changes were observed in lipogenesis in adipocytes isolated from cancer patients’ SAT, compared to controls [16]. Lower plasma TG and higher glycerol and FFAs have been observed in cachectic patients [16,17,27] but the activity or expression of LPL was not determined in these studies. Further studies are required to determine the lipogenesis capacity and fatty acid uptake by adipose tissue in various groups of cancer patients at different stages during disease trajectory.

### 4.4. Adipogenesis

Fat loss may arise from impairment in the adipose tissue development and ability for fat synthesis and storage capacity. Adipogenesis is a highly regulated process which encompasses preadipocyte proliferation and differentiation into mature adipocytes. Adipogenesis is then followed by lipogenesis to store lipid in fat cells. TNF-α, a proinflammatory cytokine produced by both tumor and adipose tissue regulates adipocyte differentiation [66]. Therefore, higher production of TNF-α in cancer-associated cachexia may lead to the altered differentiation status of adipocytes. A reduction in mRNA levels of adipogenic transcription factors including CCAAT-enhancer-binding proteins (c/EBPβ), PPARγ, c/EBPα, sterol regulatory element binding protein-1c (SREBP-1c) in epididymal adipose tissue of mice bearing MAC16 tumor was associated with diminished adipocytes size [11]. Expression of adipogenic factors including C/EBPα, SREBP1C and PPARγ decreased in rats bearing Walker 256 during early stages of cachexia. Morphological changes evident by smaller adipocytes occurred during late stages of cachexia which supports a reduction in expression of adipogenic genes [14]. Lower expression of adipogenic genes such as C/EBPα, Reverba, Per2 and PPARγ has been reported in cachectic mice bearing the Colon-26 carcinoma [62]. More research in both animal and human models is required to demonstrate the possible alterations in adipogenesis during cancer progression.

## 5. Local Adipose Tissue Inflammation

Proinflammatory cytokines, *i.e.*, IL-1β, TNF-α, IL-6 produced by tumor or host tissue due to tumor presence leads to both systemic and local inflammation in cancer [67,68]. Visceral adipose tissue is a more active producer of inflammatory cytokines IL-6, TNF-α [69,70]. However, data on local adipose tissue inflammation in cancer are inconsistent, being reported as either increased [14,62,71] or unchanged [11,16,17,31,32]. Walker 256 carcinoma caused elevated expression of macrophage markers (f4/80, CD68) especially during late stages of tumor progression [14]. Mesenteric and epididymal adipose tissue were the most and least commonly affected fat depots by macrophages, respectively [14]. In mice bearing Colon-26 carcinoma, depleted fat mass was associated with enhanced inflammatory IL-6/STAT3 cytokine signaling pathway [62]. IL-6 induces signal transducer and activator of transcription-3 (STAT3) activation through phosphorylation. Elevated levels of phosphorylated STAT3 in cachectic mice compared to the control group were observed [62]. Higher mRNA expression of TNF-α in SAT, not VAT, of cachectic GI cancer patients compared to weight-stable cancer patients was reported in newly diagnosed cancer patients. [71]. Contrary to those studies, no change in mRNA expression of inflammatory markers including IL-6 and TNF-α were observed in SAT from cancer patients [16,17] or in an animal model [11]. This paralleled the observation that macrophages or lymphocytes did not infiltrate SAT, as there were no changes in mRNA levels of CD68 (macrophages infiltration marker), CD3 (T-lymphocytes marker) [16] in humans, and MAC1 and F4/80 (macrophage markers) expression in animals [11]. Monocyte chemoattractant protein-1 (MCP-1) and TNF-α mRNA levels in both intra-abdominal and subcutaneous depots did not differ between pancreatic cancer patient and non-cancer controls. However, mRNA expression of MCP-1 and TNF-α in intra-abdominal adipose tissue negatively correlated with post-operative change in intra-abdominal mass assessed by CT scans [32]. This paradox may be due to differences in tumor stages between studies, involvement of other cytokines such as transforming growth factor-b (TGF-b), IL-1 or interferon gamma in cancer associated cachexia or the balance between anti- and pro-inflammatory cytokines of might be important for cachexia-associated inflammation [61,72]. Another explanation is that a cytokine like TNF-α is involved in early stages of cachexia but is transient in nature. Therefore, due to its short half-life and different assay sensitivities, results should be interpreted with caution (reviewed by Das and Hoefler) [61]. Alternate markers such as TNF-R1 and TNF-R2 (Soluble TNF-α membrane receptors) may be more accurate markers than TNF-α due to their longer half-life and stability [73]. Overall, a major gap remains related to comparison of local inflammatory markers in both visceral and subcutaneous depots. Inflammatory cytokines can mediate fat loss in cancer (reviewed by Bing [74]), therefore, assessing whether depot-specific differences in inflammatory cytokines transcription may contribute to inflammatory factors production and subsequent alterations in fat mass would be of great value.

## 6. Conclusions

Alterations in adipose tissue fat metabolism including changes in expression of genes involved in fat synthesis, storage, mobilization or oxidation, browning of white adipose tissue, adipocytes development, and elevated inflammatory signaling may have a role in fat loss in cancer patients. Fat accumulation at the time of diagnosis may contribute to cancer progression but the accelerated rate of adipose tissue loss would be expected to be associated with shorter survival time during cancer progression. Alterations in fat mass and composition between visceral and subcutaneous depots are equivocal in cancer trajectory and little is known regarding these alterations. The prognostic significance of these depots needs to be investigated in large populations throughout the cancer progression. Due to various roles of adipose tissue in controlling human metabolism, further identification of mechanisms and mediators of fat loss in cancer would help in the identifying fat-losing cancer patients that would benefit from early therapeutic interventions which could improve survival and prevent muscle atrophy in these patients. Finally, results need to be interpreted carefully as factors including tumor type, cancer stage, response to treatment and metabolic capacity of patients may influence findings.
